# Reviewed article: Carvalho JB, Salviano AF, Lourenço BH, Tomita LY. Temporal trend of food consumption indicators of adolescents monitored by Primary Health Care services of the Brazilian National Health System, 2015-2019. Epidemiol Serv Saude. 2025:34;e20240893

**DOI:** 10.1590/S2237-96222025v34e20240893.a

**Published:** 2025-08-04

**Authors:** Deise Warmling

**Affiliations:** Universidade Federal de Santa Catarina, Saúde Pública

**Table:** 

Rating	Excellent	Good	Average	Below average	Poor
Interest	✓				
Quality	✓				
Originality	✓				
Overall	✓				

**Table:** 

Questionnaire	Yes	No	Not applicable
Does the manuscript contain new and significant information to justify publication?	✓		
Does the Abstract (Summary) clearly and accurately describe the content of the article?	✓		
Is the problem significant and concisely stated?	✓		
Are the methods described comprehensively?	✓		
Are the interpretations and conclusions justified by the results?	✓		
Is adequate reference made to other work in the field?	✓		

## Manuscript Structure

Length of article is: Adequate

Number of tables is: Adequate

Number of figures is: Adequate

Please state any conflict(s) of interest that you have in relation to the review of this paper (state “none” if this is not applicable): None

## Comments

O artigo analisa dados de consumo alimentar, que são fundamentais para a compreensão e monitoramento do estado nutricional da população brasileira. A faixa etária analisada é subpesquisada no âmbito da APS, visto que tem baixo acesso a este nível de atenção. A baixa cobertura do SISVAN foi assumida enquanto limitação de estudo, contudo destaca-se a relevância de analisar os dados existentes e a importância do SISVAN, tal como o presente estudo fez. As análises estatísticas foram empregadas adequadamente e os resultados descritos com a clareza. O agrupamento dos indicadores em alimentos saudáveis e não saudáveis facilitou a compreensão do leitor. Considera-se o estudo relevantes para publicação e sugere-se observar os pontos a seguir:

Introdução:

linha 10 - rever termo “ocidentalizada”, pois dietas tradicionais também são ocidentais.

Discussão

Pg 6 - linha 48-58 - revisar e deixar mais clara a relação dos estudos trazidos com os resultados encontrados na presente pesquisa.

Pg 7, linha 4 - AUP aparece pela 1a vezes, deixar por extenso

Pg 7, linha 40-44 - rever redação da frase, está confusa

